# Tetrapyrrolic Macrocycles: Synthesis, Functionalization and Applications 2018

**DOI:** 10.3390/molecules25030433

**Published:** 2020-01-21

**Authors:** Nuno M. M. Moura, Maria Amparo F. Faustino, Maria Graça P. M. S. Neves

**Affiliations:** LAQV-REQUIMTE and Chemistry Department, University of Aveiro, 3810-193 Aveiro, Portugal

**Keywords:** porphyrins, corroles, phthalocyanines, BODIPY, synthetic methodologies, photophysical properties, supramolecular chemistry, catalysis

## Abstract

Natural and synthetic macrocycles like porphyrins, corroles and phthalocyanines are considered strong candidates to be used in different fields, such as catalysis, sensing, medicine, materials science, or in the development of advanced biomimetic models. All these applications are strongly dependent on the availability of compounds with adequate and specific structural features. This Special Issue has collected 13 contributions which consolidate and expand our knowledge on the application of these macrocycles in different fields accompanied by innovative synthetic methodologies to afford and to functionalize this type of compounds.

Porphyrins and analogues show special features to be used in different scientific areas like catalysis, sensing, medicine, development of advanced biomimetic models, and materials science. It is recognized that the success of these compounds is related with the availability of compounds with adequate structural features for a specific application. The efforts of synthetic researchers in developing new methodologies to prepare and to functionalize these macrocycles as well as to assess their photochemical and biological properties are responsible for their recognized success. This Special Issue collects 13 contributions which consolidate and expand our knowledge on the application of these macrocycles in different fields and on innovative synthetic approaches.

Considering that porphyrins and fullerenes are attractive building blocks in the design of supramolecular assemblies, Tomé and co-workers report a selective and efficient synthetic access to diporphyrins and pentaporphyrins through the reaction of 5-(4-hydroxyphenyl)-10,15,20-tri phenylporphyrin with 5,10,15,20-tetrakis(pentafluorophenyl)porphyrin or with its Zn(II) complex [1]. The authors verified that the four free-base porphyrin units present in the pentaporphyrin coordinated with Zn (II) have an important contribution in the stabilization of the complex obtained during titrations with a pyridyl[60]fulleropyrrolidine derivative presumably due to a synergic effect between the fullerene unit and the four free-base porphyrin units mediated by π–π interactions.

The importance of porphyrins bearing nitro groups as potential anticancer agents and also as templates for further transformations to target moieties of higher complexity prompted Agnieszka Mikus and co-workers to revisit the reaction between Cu(II), Ni(II), and Co(II) metallocomplexes of 5,10,15,20-tetraphenylporphyrin with diluted nitric acid [2]. The authors found that the nitration can be carried out selectively to give mainly β,β-dinitro-compounds in yields up to 73%, whose structures were assigned based on NMR studies.

Considering that telomerase is a reverse transcriptase enzyme overexpressed in a range of cancer cells that allows continuous cell division without telomere shortening makes this enzyme an attractive therapeutic target. Under this context, the development of ligands able to stabilize selectively G-Quadruplex DNA structures over duplex DNA is considered a promising cancer therapy approach. Ramos and co-workers [3] using different spectroscopic techniques, found that the phthalocyanines with four positive charges and with less exposed eight positive charges showed high selectivity and affinity for G-Quadruplex over duplex DNA structures and were able to accumulate in the nucleus of UM-UC-3 bladder cancer cells.

It is well established that a high number of diseases are usually associated with long periods of exposure to potentially toxic metals ions. Considering the high ability of porphyrins to coordinate metal ions, Radi and co-workers report the preparation and characterization of a hybrid material obtained from the immobilization of 5,10,15,20-tetrakis(pentafluorophenyl)porphyrin on silica surface and studied its efficacy to remove Pb(II), Cu(II), Cd(II), and Zn(II) ions from water [4]. The study also shows that metal cations from real river water samples can be efficiently removed in the presence of the new adsorbent material and in terms of mass the highest adsorption capacity was found for Pb(II).

The synthetic chemistry of corroles is much less developed when compared with porphyrins and in this special issue, Osuka and co-workers contributed with the first synthetic access to *meso*-diarylaminocorroles [5]. The strategy involved the nucleophilic aromatic substitution of a *meso*-chlorocorrole silver complex with diphenylamine or carbazole. The optical and electrochemical measurements allowed concluding that the electronic states of the *meso*-diphenylaminocorrole are more strongly perturbed when compared with the corrole bearing the carbazole unit. Attempts to oxidize the *meso*-diphenylaminocorrole with 2,3-dichloro-5,6-dicyano-1,4-benzoquinone in ethanol afforded an unexpected isocorrole substituted with two ethoxy units at the *meso* position.

Shaya Y. Alraqa and co-workers reported a synthetic strategy to afford the 1,4,8,11,15,18,22,25-octahexyloxy-2,3,9,10,16,17,23,24-octakis(4-trifluoromethoxyphenyl)phthalocyanine and the corresponding zinc(II) complex which showed to be soluble in most organic solvents [6]. The 4-trifluoromethoxyphenyl groups were introduced by a Suzuki-Miyaura coupling reaction and the hexyloxy groups by the Mitsunobu reaction at the phthalonitrile stage using the 4,5-dibromo-3,6-dihydroxyphthalonitrile. The photophysical and photochemical properties show that both phthalocyanines can be considered promising photosensitizers for Photodynamic Therapy (PDT).

Mariette Pereira and co-workers contributed to this issue, by reporting the epoxidation of (−)-isopulegol benzyl ether using molecular oxygen as oxidant, isobutyraldehyde as co-reductant and a hybrid Mn(III)-porphyrin magnetic nanocomposite as the catalyst [7]. The authors found that the magnetic catalyst can be reused efficiently at least four times. The anticancer profile of the obtained epoxides evaluated on a human osteosarcoma cell line (MG-63) showed that both diastereomers have a similar in vitro profile and present IC_50_ values significantly lower than the ones reported for other monoterpene analogues.

Recent therapeutic strategies are based on activatable photosensitizers which are molecules that restore their activities in tumor microenvironments due to removal of sensitive linkers between the quencher and the photosensitizer and are being considered as having relevance for new PDT developments. Under this context, Jian-Yong Liu and co-workers report the synthetic access to a boron dipyrromethene (BODIPY) bearing iodine atoms and an arylboronate group that quenches the excited state of the BODIPY photosensitizer by a photoinduced electron transfer process [8]. However, the BODYPY in the presence of H_2_O_2_ can restore the fluorescence emission and singlet oxygen production due to the cleavage of the arylboronate group. The study shows that due to the higher concentration of H_2_O_2_ in cancer cells the BODYPY is particularly sensitive to human cervical carcinoma (HeLa) cells without damaging human embryonic lung fibroblast (HELF).

A convenient protocol to obtain the water-soluble porphyrin 5-phenyl-10,15,20-tris (4-sulfonatophenyl) porphyrin (TPPS_3_) in gram scale is described by Albert Mayano and co-workers [9]. The procedure relies on the one-pot reductive deamination of 5-(4-aminophenyl)-10,15,20-tris (4-sulfonatophenyl)porphyrin. The authors evaluate the efficacy of the zwitterionic aggregates derived from TPPS_3_ and cyclic secondary amines as a supramolecular catalyst in a Diels–Alder reaction between cinnamaldehyde and cyclopentadiene performed in aqueous medium.

Simone and co-workers use the density functional theory (DFT) to predict the photophysical properties of a series of nitrated and halogenated phosphorus tritolylcorrole complexes in dichloromethane to give insights into their possible use as photosensitizers in PDT [10]. The analysis shows that the of iodine atoms as peripheral substituents increase significantly the spin-matrix elements which allow concluding that phosphorus tritolylcorrole complexes containing iodine can be suggested as photosensitizers in PDT.

Rodrigues and co-workers reported a new methodology to introduce thioaryl moieties in the 5,10,15,20-tetrakis(pentafluorophenyl) porphyrin core via a reductive cleavage of disulfides [11]. The evaluation of their photophysical parameters allows concluding that the thioaryl-porphyrin hybrids have potential to be used as photosensitizers for PDT. The experimental and theoretical DNA interactive assays also performed, confirm the existence of effective interaction between the porphyrinic hybrids and DNA.

In this special issue, there is also the contribution of two reviews [12,13]. In the one from H.-Y. Liu and co-workers it is summarized the developments to identify high-valent cobalt-oxo species of tetrapyrrolic macrocycles and *N*-based ligands [12]. These oxo species are involved in cobalt-oxo complexes preparation, organic substrates oxidation, and water oxidation reactions and their knowledge have an important contribution to understanding the mechanism of oxygen evolution in photosynthesis and can help to mimic enzymatic catalysis in non-natural systems.

In the other review, S. Nakagaki and co-workers discuss the ability of porphyrins to act as multifunctional catalysts in sequential one-pot reactions, affording the desirable organic products in just one step [13]. The authors give special attention to oxidation reactions using molecular dioxygen as terminal oxidant; photoredox reactions; oxidation-isomerization; epoxidation-ring opening; oxidation-acetalization; *N*-H insertion in carbenes; oxidation of 1,2-diones to produce diacids, ketoacids and diketoacids; and production of HNO.
